# Crisis averted: a world united against the menace of multiple drug-resistant superbugs -pioneering anti-AMR vaccines, RNA interference, nanomedicine, CRISPR-based antimicrobials, bacteriophage therapies, and clinical artificial intelligence strategies to safeguard global antimicrobial arsenal

**DOI:** 10.3389/fmicb.2023.1270018

**Published:** 2023-11-30

**Authors:** Umar Saeed, Rawal Alies Insaf, Zahra Zahid Piracha, Muhammad Nouman Tariq, Azka Sohail, Umer Ali Abbasi, Muhammad Shahmeer Fida Rana, Syed Shayan Gilani, Seneen Noor, Elyeen Noor, Yasir Waheed, Maryam Wahid, Muzammil Hasan Najmi, Imran Fazal

**Affiliations:** ^1^Clinical and Biomedical Research Center (CBRC) and Multidisciplinary Laboratories (MDL), Foundation University School of Health Sciences (FUSH), Foundation University Islamabad (FUI), Islamabad, Pakistan; ^2^Regional Disease Surveillance and Response Unit Sukkur, Sukkur, Sindh, Pakistan; ^3^International Center of Medical Sciences Research (ICMSR), Islamabad, Pakistan; ^4^Akhtar Saeed Medical and Dental College (AMDC), Lahore, Pakistan; ^5^Central Park Teaching Hospital, Lahore, Pakistan; ^6^Office of Research, Innovation, and Commercialization (ORIC), Shaheed Zulfiqar Ali Bhutto Medical University, Islamabad, Pakistan; ^7^Gilbert and Rose-Marie Chagoury School of Medicine, Lebanese American University, Byblos, Lebanon

**Keywords:** antimicrobial resistance, bacterial infections, virus-like particles, phage-based therapies, vaccine development, multiple drug-resistant species, artificial intelligence

## Abstract

The efficacy of antibiotics and other antimicrobial agents in combating bacterial infections faces a grave peril in the form of antimicrobial resistance (AMR), an exceedingly pressing global health issue. The emergence and dissemination of drug-resistant bacteria can be attributed to the rampant overuse and misuse of antibiotics, leading to dire consequences such as organ failure and sepsis. Beyond the realm of individual health, the pervasive specter of AMR casts its ominous shadow upon the economy and society at large, resulting in protracted hospital stays, elevated medical expenditures, and diminished productivity, with particularly dire consequences for vulnerable populations. It is abundantly clear that addressing this ominous threat necessitates a concerted international endeavor encompassing the optimization of antibiotic deployment, the pursuit of novel antimicrobial compounds and therapeutic strategies, the enhancement of surveillance and monitoring of resistant bacterial strains, and the assurance of universal access to efficacious treatments. In the ongoing struggle against this encroaching menace, phage-based therapies, strategically tailored to combat AMR, offer a formidable line of defense. Furthermore, an alluring pathway forward for the development of vaccines lies in the utilization of virus-like particles (VLPs), which have demonstrated their remarkable capacity to elicit a robust immune response against bacterial infections. VLP-based vaccinations, characterized by their absence of genetic material and non-infectious nature, present a markedly safer and more stable alternative to conventional immunization protocols. Encouragingly, preclinical investigations have yielded promising results in the development of VLP vaccines targeting pivotal bacteria implicated in the AMR crisis, including *Salmonella, Escherichia coli,* and *Clostridium difficile*. Notwithstanding the undeniable potential of VLP vaccines, formidable challenges persist, including the identification of suitable bacterial markers for vaccination and the formidable prospect of bacterial pathogens evolving mechanisms to thwart the immune response. Nonetheless, the prospect of VLP-based vaccines holds great promise in the relentless fight against AMR, underscoring the need for sustained research and development endeavors. In the quest to marshal more potent defenses against AMR and to pave the way for visionary innovations, cutting-edge techniques that incorporate RNA interference, nanomedicine, and the integration of artificial intelligence are currently under rigorous scrutiny.

## Introduction

The effectiveness of antibiotics and other antimicrobial drugs in the treatment of bacterial infections is under threat due to antimicrobial resistance (AMR), a problem that has become a worldwide public health concern. AMR happens when bacteria become resistant to the medications that are meant to eradicate or slow down their growth, leading to greater complexity in managing infections and heightened chances of severe outcomes, such as organ failure and sepsis (Ferri et al., 2017; Karimi et al., 2023). The rise in antibiotic usage and misuse has resulted in the emergence and spread of drug-resistant bacteria, thereby making AMR a significant worldwide health hazard in recent times. The World Health Organization states that AMR is included in the list of the most pressing global health hazards and that around 700,000 fatalities are caused annually as a result of AMR-related illnesses (World Health Organization, 2023). The consequences of AMR go beyond just human health, having a noteworthy impact on both the economy and society. AMR can result in extended hospitalization, escalated medical expenditures, and reduced efficiency, ultimately causing severe consequences on susceptible communities, including children, senior citizens, and individuals with compromised immunity. A well-planned and synchronized worldwide effort is required to confront the danger of AMR. In order to combat the rise of antibiotic resistance, measures such as optimizing antibiotic use, promoting research into new antimicrobial drugs and therapies, and enhancing surveillance and monitoring methods to detect the spread of resistant bacteria, are being implemented (Larsson and Flach, 2022). Additionally, it is imperative that there is a worldwide collaboration and alliances to furnish financial backing for research and innovation, enhance proficiency in managing antimicrobial usage, infection prevention, and restrain its spread, as well as guarantee availability and accessibility to potent antimicrobial remedies to all people irrespective of their geographical or economic status (Bump et al., 2021).

Acknowledged as a formidable global health challenge, communicable diseases have long cast a shadow over public well-being, contributing to more than 20% of global deaths in 2017. Among these health threats, bacterial infections have emerged as a clinically significant and widespread cause of health loss across the globe. Despite their profound impact, a critical gap in our understanding exists regarding the comprehensive assessment of the global burden posed by many common bacterial pathogens. This gap has hindered the establishment of effective public health priorities, leaving us with fragmented estimates often limited to specific pathogens, select populations, or affluent nations (Larsson and Flach, 2022).

Notably, the plight of low-income and middle-income countries (LMICs), where the burden of infectious diseases is most pronounced, has been largely sidelined in global advocacy efforts. These regions face unique challenges, including limited access to healthcare resources, inadequate sanitation and hygiene, and a lack of vaccination programs. This exacerbates the burden of bacterial infections, resulting in a disproportionate impact on vulnerable populations, particularly young children under the age of five. Alarmingly, one in five deaths in this age group is attributed to once-treatable infections, such as vaccine-preventable pneumococcal bacterial disease. Bacterial antimicrobial resistance (AMR) has surged to the forefront of global health threats. AMR now threatens to surpass even the toll of HIV/AIDS and malaria by 2050 if left unchecked (Ho and Ip, 2019; Bump et al., 2021). The ramifications are staggering, with the mortality associated with AMR already surpassing that of these high-profile diseases. This looming crisis necessitates immediate attention and concerted efforts to address the root causes, such as the overuse and misuse of antibiotics in healthcare and agriculture. Further emphasizing the urgency of the situation, a handful of bacterial pathogens have taken center stage in the global mortality landscape. Five bacterial pathogens – *Staphylococcus aureus*, *Escherichia coli*, *Streptococcus pneumoniae*, *Klebsiella pneumoniae*, and *Pseudomonas aeruginosa* – collectively accounted for over half of all global bacterial deaths. These pathogens span a range of infectious syndromes, from respiratory and urinary tract infections to sepsis, causing significant morbidity and mortality (Ho and Ip, 2019).

To tackle this substantial burden, a multifaceted approach is required. This approach encompasses strengthening healthcare systems in LMICs, improving diagnostics to enable early and accurate identification of bacterial infections, enhancing infection control practices in healthcare settings, and promoting prudent antimicrobial stewardship to curb the spread of AMR. Preventive strategies are also paramount, including initiatives to improve access to safe drinking water, sanitation facilities, and vaccination. The development of new vaccines and improved access to appropriate antibiotics for infections are critical components of this effort. Balancing the right to antimicrobial access with responsible usage, particularly for expensive and newer-generation antimicrobials, is a delicate and crucial task. Overuse and misuse of antibiotics have contributed significantly to the rise of AMR, and effective policies and guidelines are needed to address this issue.

Extensively Drug-Resistant (XDR) pathogens epitomize a critical and alarming subset of antibiotic-resistant bacteria, demonstrating unparalleled adaptability by acquiring resistance to multiple classes of antibiotics, even those deemed as the last resort in our therapeutic armamentarium. This classification of bacteria poses a grave and imminent threat to public health, as it severely constricts the therapeutic options available for combatting the infections they instigate. Notable XDR pathogens include *Acinetobacter baumannii*, *Pseudomonas aeruginosa*, *Klebsiella pneumoniae*, *Enterobacter* spp., *Escherichia coli*, and *Staphylococcus aureus*, among which the MRSA, stands prominently. These microorganisms have earned notoriety for their extraordinary capability to develop resistance mechanisms at a rate that frequently outpaces the development of novel antibiotics. Consequently, healthcare providers find themselves confronted with the formidable challenge of managing infections caused by these highly resistant bacteria, armed with a rapidly dwindling arsenal of effective therapeutic agents. XDR infections can manifest across diverse healthcare settings, ranging from hospital environments to long-term care facilities, underscoring their profound implications for patient safety (Larsson and Flach, 2022).

On the other hand, Pan-Drug-Resistant (PDR) pathogens represent the most dire and distressing category of antibiotic-resistant bacteria. In contrast to XDR bacteria, PDR strains exhibit resistance to nearly all available antibiotics, leaving healthcare providers with exceptionally restricted or non-existent treatment alternatives. Prominent PDR pathogens encompass specific strains of *Acinetobacter baumannii*, *Pseudomonas aeruginosa*, and *Enterobacter* spp., among others. Infections induced by PDR pathogens present formidable challenges, yielding dire consequences for patient outcomes and straining healthcare systems. Healthcare providers often resort to supportive care and symptom management when faced with PDR infections, as there are scant to no effective antimicrobial therapies available. These infections are associated with alarmingly high mortality rates, protracted hospitalization periods, and escalated healthcare expenditures. The emergence and dissemination of PDR pathogens underscore the pressing imperative for enhanced infection control measures, meticulous antimicrobial stewardship initiatives, and the imperative pursuit of novel antibiotic development. Furthermore, fostering international collaboration and data sharing is of paramount significance in the endeavor to monitor and combat the burgeoning global burden imposed by PDR infections. Effectively addressing the grave threats posed by PDR pathogens mandates the implementation of a multifaceted approach, one that prioritizes public health protection and seeks to mitigate the dire consequences ensuing from antibiotic resistance (Ho and Ip, 2019).

Vancomycin-Resistant *Staphylococcus aureus* (VRSA) signifies a formidable and alarming advancement in the realm of antimicrobial resistance, engendering deep-seated concerns within the global healthcare landscape. *Staphylococcus aureus*, colloquially known as Staph aureus, stands as a remarkably versatile bacterium, capable of instigating a spectrum of infections, spanning from minor skin maladies to life-threatening bloodstream afflictions. The emergence of VRSA introduces a profound layer of complexity into the relentless battle against antibiotic-resistant pathogens. VRSA strains are characterized by their remarkable ability to withstand vancomycin, a stalwart of antimicrobial therapies and a cherished last-line defense against severe Staph aureus infections, including the notorious MRSA (World Health Organization, 2023). This resistance stems from the acquisition of specific genetic components, such as the vanA gene, which empowers the bacterium to orchestrate modifications to its cell wall, rendering vancomycin ineffectual in its combat. The ramifications of VRSA are profound and far-reaching. They substantially curtail the array of treatment avenues available for infections, potentially culminating in protracted hospitalizations, heightened healthcare expenditures, and an ominous elevation in mortality rates. Healthcare environments, notably hospitals and long-term care facilities, stand as fertile grounds for the dissemination of VRSA, accentuating the paramount importance of stringent infection prevention and control measures (Larsson and Flach, 2022). Effectively addressing the menace of VRSA necessitates the adoption of a multi-pronged strategy. This encompasses vigilant surveillance, unwavering adherence to infection control protocols, judicious employment of antibiotics, and sustained exploration of alternative therapeutic modalities. Moreover, the development of novel antibiotics, underpinned by innovative mechanisms of action, assumes pivotal significance in the endeavor to combat VRSA and its resilient companions among antibiotic-resistant pathogens. In the face of VRSA’s menacing emergence and the overarching challenge posed by antimicrobial resistance, unwavering vigilance, collaborative synergy, and inventive solutions stand as imperatives (Bump et al., 2021). By furthering our comprehension of VRSA and deploying comprehensive strategic frameworks, we endeavor to curtail its propagation, safeguarding our capacity to effectively combat Staph aureus infections on a global scale.

In the unyielding quest to combat antimicrobial resistance (AMR) and confront the formidable specter of multiple drug-resistant bacterial, viral, and fungal adversaries, we embark on a visionary odyssey defined by cutting-edge innovation and global collaboration. We envision precision nanomedicine, where advanced nanotechnology-based drug delivery systems navigate with unparalleled precision to the heart of infections, mitigating resistance risks while sparing healthy cells. CRISPR-Cas gene editing empowers us to disarm drug resistance genes at the microbial genetic level, crafting personalized therapies for each patient. Artificial intelligence and machine learning expedite the discovery of novel antimicrobial compounds, reshaping drug development. Phage-based therapies and sentinel networks redefine our defense strategies. Antibiotic stewardship, pan-viral antivirals, synergistic combinations, microbiome rejuvenation, innovative vaccination, global awareness initiatives, and a “One Health” imperative fortify our arsenal. This harmonious symphony of futuristic approaches promises to revolutionize our battle against AMR, safeguarding global health from ever-evolving pathogens. The application of predictive mathematical modeling and advancements in genomic epidemiology holds promise in better understanding pathogen evolution, epidemiology, and pathogenesis at a global level. These tools can inform future approaches to tackle this burgeoning crisis and guide the development of targeted interventions. Comprehensive estimates of bacterial infection mortality, coupled with a concerted global effort, are imperative to inform strategies aimed at reducing the burden of bacterial infectious diseases and addressing the escalating threat of antimicrobial resistance. This challenge requires a coordinated response, involving governments, healthcare systems, researchers, and international organizations, to safeguard global public health.

### Antimicrobial resistance looms as a formidable threat to the well-being of the entire global populace

Several typical microorganisms result in resistance to antimicrobial drugs. Antimicrobial resistance (AMR) is a concern that can manifest across various pathogens, encompassing bacteria, fungi, viruses, and parasites. However, it is in the realm of bacteria where AMR predominantly takes root. One of the archetypal bacterial culprits fostering AMR is *Escherichia coli*, a ubiquitous bacterium known for its role in ailments like food poisoning and urinary tract infections. This microorganism frequently resides in the human and animal intestinal tracts and can be transmitted through tainted food or water sources. While *E. coli* is a customary inhabitant of the human digestive system, certain strains have the potential to trigger severe conditions such as UTIs, bloodstream infections, and pneumonia. The emergence of antimicrobial resistance in East. coli represents a pivotal concern, as it stands prominently among the leading catalysts of AMR infections worldwide (Ho and Ip, 2019). *Staphylococcus aureus* (*S. aureus*) is a type of bacteria that causes a wide range of infections in humans. It is commonly found on the skin and in the nasal passages, and can cause illnesses ranging from minor skin infections to life-threatening conditions such as pneumonia, sepsis, and endocarditis. *S. aureus* is becoming increasingly resistant to antibiotics, making it a serious public health concern worldwide. Researchers are actively working to develop new treatments and prevention strategies to combat this dangerous bacterium. The *S. aureus* bacterium is often present in the skin and nasal cavity but can result in various infections such as skin infections, pneumonia, and infections of the bloodstream. Several antibiotics are ineffective against aureus, particularly *Methicillin Resistant Staphylococcus aureus* (MRSA), due to its resistance. *Klebsiella pneumoniae*, a type of bacteria frequently present in the gastrointestinal tract, has the potential to induce various illnesses including pneumonia, urinary tract infections, and bloodstream infections. Antimicrobial resistance in *K. pneumoniae* poses a significant threat due to its multi-drug resistant nature, including resistance to carbapenems that are typically reserved for severe infections as a last resort (McNally et al., 2019). *Acinetobacter baumannii* is a bacteria often detected in medical facilities that can lead to infections like bloodstream infections and pneumonia. *A. baumannii* poses a grave threat due to certain strains being unresponsive to various antibiotics, including carbapenems. The bacterium known as *Pseudomonas aeruginosa* is frequently present in healthcare environments and has the potential to bring about infections like bloodstream infections and pneumonia. *Pseudomonas aeruginosa* poses a significant threat since certain variations exhibit resistance to numerous antibacterial medications, such as carbapenems. Aside from the usual bacterial pathogens, fungi, and viruses also have the ability to become resistant to antimicrobial medications, resulting in infections that are challenging to manage. The occurrence and dissemination of AMR are intricate and diverse issues that demand a unified effort from multiple sectors to resolve (Anjum et al., 2017).

### Innovative phage-based therapies aimed at combating the scourge of antimicrobial resistance

The use of bacteriophages, which are viruses that attack and eliminate bacteria, set the groundwork for an innovative approach called bacteriophage therapy in treating bacterial infections. The use of bacteriophage therapy has regained attention as a promising substitute for antibiotics, especially in the face of AMR (Usman et al., 2023). The application of bacteriophages in combating bacterial infections is not a novel idea, and the origins of research on bacteriophage therapy can be traced back to the beginning of the 1900s. The excessive use of antibiotics caused a decrease in the fascination toward bacteriophage therapy which ultimately was disregarded in Western nations in preference to antibiotics (Huang et al., 2022). The rise of AMR has reignited curiosity in bacteriophage therapy as a potential substitute for antibiotics in recent times. Bacteriophages possess a remarkable ability to infect and eliminate bacteria with precision, and they can be customized to attack particular strains or species, culminating in a hopeful alternative for treating infections that emerge from antibiotic-resistant bacteria. Bacteriophage therapy has a significant benefit in its capacity to adjust and develop in accordance with variations in bacterial communities. As bacteriophages become less effective on resistant bacteria, novel phages can be identified and employed to specifically target these adaptive strains (Carascal et al., 2022). The potential effectiveness of bacteriophage therapy in combating AMR lies in its capability to adjust and develop over time. Bacteriophage therapy has proven to be an effective treatment for various bacterial infections such as those affecting the skin, gastrointestinal tract, and urinary tract. Despite the potential benefits of bacteriophage therapy, there are certain obstacles that need to be addressed in order for it to be widely acknowledged and integrated as a solution for bacterial infections (Bhattacharjee et al., 2022). There are several obstacles to overcome such as meeting regulatory requirements, ensuring consistent production and quality, and conducting additional clinical tests to confirm safety and effectiveness. Furthermore, it is imperative to increase knowledge and understanding of bacteriophage therapy among both healthcare professionals and the general population (Khan et al., 2022; Wu et al., 2022).

### Multi-drug resistant bacterial pathogens

Multidrug-resistant (MDR) microorganisms pose a considerable risk to public health since they can result in severe infections, especially among people with weakened immune systems, and are challenging to cure. Several widespread bacterial pathogens that are resistant to multiple drugs (MDR) include; MRSA which cannot be treated with various types of antibiotics, including beta-lactams (Vestergaard et al., 2019). MRSA is a prevalent cause of infections related to healthcare facilities, including bloodstream infections, pneumonia, and surgical site infections. ESBL-producing bacteria, such as *Escherichia coli* and *Klebsiella pneumoniae*, belong to the family of Enterobacteriaceae, which comprises many known pathogens. ESBL-producing Enterobacteriaceae pose a challenge in terms of treatment due to their resistance to a variety of antibiotics such as beta-lactams and frequently carbapenems (Khawbung et al., 2021). *Acinetobacter baumannii*, frequently found in medical facilities, plays a crucial role in causing infections such as bloodstream infections and pneumonia. *Baumannii*, a type of strain, possesses resistance against various antibiotic classes, which encompasses carbapenems. *Pseudomonas aeruginosa*, often encountered in healthcare facilities, is a primary culprit behind infections such as bloodstream infections and pneumonia (Wang et al., 2020). *Pseudomonas aeruginosa* strains exhibit resistance to several types of antibiotics, including carbapenems. *Mycobacterium tuberculosis* is responsible for causing tuberculosis, which poses a significant health hazard worldwide. MDR TB is a form of TB that cannot be treated with two of the primary antibiotics, namely isoniazid and rifampicin (Liu et al., 2021).

### The specter of antibiotic resistance casts its shadow across all seven continents of our global landscape

The issue of Antimicrobial resistance (AMR) poses a significant risk to public health worldwide, impacting every region across the globe. The worldwide dilemma of antimicrobial resistance and the occurrence of AMR strains in North America. AMR is a significant issue related to public health in North America, as high levels of resistance have been found in various bacterial pathogens such as MRSA and Carbapenem Resistant Enterobacteriaceae (CRE). The issue of antimicrobial resistance (AMR) poses a grave danger to global public health, with an estimated 700,000 deaths per year being caused by infections resulting from AMR. The increasing prevalence of AMR in North America is becoming a cause of worry, as several bacterial types have shown a significant level of resistance (McDonald et al., 2019). The Centers for Disease Control and Prevention (CDC) in the US have approximated that around 2.8 million people get infected annually with bacteria that are resistant to antibiotics, leading to the demise of more than 35,000 people. In the United States, the prevalent AMR types are MRSA, CRE, and vancomycin-resistant Enterococci (VRE) (Dodds, 2017).

In Canada, AMR is a major issue with reports suggesting a rise in resistance among a number of bacterial strains such as MRSA, *Escherichia coli, and Klebsiella pneumoniae*. The rates of antimicrobial resistance (AMR) to commonly prescribed antibiotics are reportedly rising across Canada as per the monitoring system known as Canadian Antimicrobial Resistance Surveillance System (CARSS) (Davies and Davies, 2010).

Mexico has divulged notable levels of antimicrobial resistance, specifically in infections that are obtained in healthcare settings. In 2018, a research revealed that antibiotic-resistant bacteria accounted for almost 40% of infections acquired in hospitals throughout Mexico, with *Pseudomonas aeruginosa*, *Klebsiella pneumoniae*, and *Acinetobacter baumannii* being the most frequently identified resistant strains. In North America, AMR poses a major threat to public health as various bacterial species have been found to exhibit significant rates of resistance. To address AMR, several strategies are being employed such as enhancing monitoring, minimizing the unwarranted utilization of antibiotics, creating novel antimicrobial treatments, and elevating awareness and instructing healthcare professionals and the general populace. The collective impact of antimicrobial resistance and the occurrence of AMR species in South America on a global level (Amabile-Cuevas, 2021).

South America is confronted with considerable difficulties concerning AMR, as several bacterial pathogens, such as MRSA and *Pseudomonas aeruginosa*, have been documented with high resistance rates. Antibiotic resistance is a significant global concern that impacts every region, including South America, as a public health hazard. The escalating issue of AMR in South America has raised alarms as numerous bacterial species depict increased rates of resistance. AMR poses a prominent public health challenge in Brazil, as studies have indicated notable resistance rates to widely utilized antibiotics within various bacterial strains, such as *Klebsiella pneumoniae*, *Pseudomonas aeruginosa*, and *Acinetobacter baumannii*. Pneumonia strains found in Brazilian hospitals have demonstrated a resistance to third-generation cephalosporins, which are frequently utilized antibiotics (Bonelli et al., 2014).

In Argentina, there have been reports suggesting that various bacterial species, such as MRSA were exhibiting elevated levels of resistance. These microorganisms are commonly referred to as *Pseudomonas aeruginosa* and *Klebsiella pneumoniae*. Vancomycin resistance in MRSA isolates from medical facilities in Argentina stood at 35%, which is concerning since vancomycin is a crucial antibiotic used as a last resort to manage severe MRSA infections. Several bacterial species have been found to have high rates of AMR in countries such as Colombia, Peru, and Chile, which are located in South America. Several countries in the region are facing a major challenge in dealing with resistance toward third-generation cephalosporins. Antimicrobial resistance is a notable menace to public health in South America, marked by increased occurrences of resistance in various bacterial types. Solving the issue of AMR in the area will necessitate a synchronized worldwide endeavor, which involves bolstering surveillance, minimizing the nonessential utilization of antibiotics, innovating novel antimicrobial remedies, and stimulating awareness and education among healthcare professionals and the general public (Moreno-Switt et al., 2019).

### The overarching ramifications of antimicrobial resistance and the prevalence of AMR strains in Europe

Europe exhibits some of the most elevated AMR statistics globally, with numerous bacterial pathogens displaying resistance, such as MRSA, VRE, and multidrug-resistant tuberculosis (MDR-TB). Antimicrobial resistance (AMR) poses a universal danger to public health, including Europe. The increasing worry in Europe is the weight of AMR, as various bacterial species show elevated rates of resistance. Around 33,000 individuals who contract antibiotic-resistant bacteria die annually in the European Union (EU), according to estimates by the European Centre for Disease Prevention and Control (ECDC). The prevalent AMR organisms in Europe consist of MRSA, *E. coli*, *K. pneumoniae*, and *A. baumannii*. The UK is facing a major issue in terms of public health as AMR continues to grow, according to reports. In the UK, a significant proportion of infections caused by *E. coli* bacteria demonstrated resistance to at least one antibiotic that is commonly prescribed (Tacconelli and Pezzani, 2019).

According to reports, numerous bacterial species, such as MRSA, *Pseudomonas aeruginosa*, and *K. pneumoniae* have exhibited elevated resistance rates in Germany. Carbapenem, which is a potent antibiotic employed to treat severe infections arising from antibiotic-resistant bacteria, was not effective against *pneumoniae* isolates obtained from German hospitals (Hassoun-Kheir and Harbarth, 2022).

Several bacterial species have been reported to have high rates of AMR in other European countries such as Spain, Italy, and France. Resistance to third-generation cephalosporins and carbapenems poses a major challenge in a number of countries within the region. Europe is facing a major health challenge due to AMR, as various bacterial species present elevated levels of resistance. Effectively tackling the issue of AMR within the locality will necessitate an integrated worldwide endeavor, encompassing upgraded monitoring systems, minimization of the avoidable administration of antibiotics, creation of fresh antimicrobial remedies, and enhancing understanding and education among medical practitioners and the general populace (Machowska and Stålsby, 2018).

### The global repercussions of antimicrobial resistance and the presence of AMR variants in Africa

AMR has become a pressing issue in Africa due to the prevalence of resistant bacterial pathogens like MRSA and *Acinetobacter baumannii*, which have been reported to exhibit high rates of multidrug resistance. The African region faces an escalating danger to public health from AMR. The prevalence of infectious diseases in Africa and the limited healthcare resources in the region make the AMR burden a cause for specific concern (Founou et al., 2018). In various bacterial species such as *Escherichia coli*, *Klebsiella pneumoniae*, *and Acinetobacter baumannii* there are significant occurrences of resistance reported within Africa (Ouedraogo et al., 2017). The African hospitals’ *pneumoniae* strains demonstrate resistance to the routinely employed third-generation cephalosporins antibiotic group. Furthermore, the prevalence of MDR-TB is a major concern in various African nations, wherein the efficacy of primary and secondary antibiotics utilized for curing TB is considerably low due to resistance (Walusansa et al., 2022; Chibwe et al., 2023). About 10% of recently detected tuberculosis cases in Africa had developed resistance toward one or more primary antibiotics. AMR has an impact on other contagious illnesses in Africa, like malaria and HIV. According to recent reports, multiple countries in the area are experiencing a rise in resistance to antimalarial medications, particularly artemisinin-containing combined therapies (ACTs), which are the most commonly used treatment for malaria. Furthermore, there are reports that suggest a rise in the prevalence of resistance to the antiretroviral medications utilized for the management of HIV (Kayode and Okoh, 2022). In Africa, AMR poses a considerable risk to public health due to numerous bacterial strains and illnesses reporting high levels of resistance. Investing in healthcare infrastructure and resources in this area is crucial to enhancing both the prevention and management of infectious diseases and antimicrobial resistance (García-Vello et al., 2020).

### The global burden of antimicrobial resistance and the prevalence of resistant species in Asia

Asia has emerged as one of the regions with the highest levels of AMR globally, as several bacterial pathogens, including; MRSA, CRE, and XDR-TB, have been found to exhibit high rates of resistance. In Asia, there is a notable concern regarding public health due to AMR. The high incidence of infectious illnesses in Asia, coupled with the extensive usage of antibiotics, is a cause for significant alarm. According to recent reports, a number of bacterial strains in Asia, specifically *Escherichia coli*, *Klebsiella pneumoniae*, and *Acinetobacter baumannii*, are exhibiting elevated levels of resistance. Carbapenem-resistant Enterobacteriaceae (CRE) is a major public health issue in the region, with numerous countries reporting high resistance levels. MDR-TB poses a significant challenge in Asia, where there are elevated levels of resistance observed toward the primary and secondary antibiotics utilized to combat tuberculosis. Around 30% of individuals newly diagnosed with tuberculosis in the area are reported to have resistance to a minimum of one primary antibiotic, while approximately 6% of global MDR-TB infections stem from India (Ha et al., 2019).

AMR is also impacting other contagious illnesses prevalent in Asia, such as dengue fever and malaria. According to reports, a growing number of countries in the area are experiencing higher levels of resistance to antimalarial medications, particularly ACTs, which are the primary means of treating malaria. Moreover, records suggest a growing trend of antiviral drug resistance in the treatment of dengue fever. AMR presents a major issue for public health in Pakistan, as multiple bacterial species have been reported to exhibit high rates of resistance. There are various factors that contribute to the worsening of the issue of antimicrobial resistance (AMR) in Pakistan. These include improper utilization of antibiotics, inadequate implementation of infection control measures, and restricted availability of effective treatments for antimicrobial agents. It has been reported that Pakistan is experiencing significant levels of bacterial resistance in a variety of species, such as *Escherichia coli*, *Klebsiella pneumoniae*, and *Acinetobacter baumannii*. *Carbapenem-resistant Enterobacteriaceae* (CRE) have become a major public health issue in the country, particularly due to their prevalent resistance in numerous hospitals (Lim, 1994).

In Pakistan, there is a major challenge posed by MDR-TB as a considerable proportion of individuals diagnosed with TB show resistance to both primary and secondary antibiotics utilized in the treatment of the condition. Newly released reports suggest that a considerable proportion – about 27% – of fresh TB patients in the region show resistance to one or more of the first-line antibiotics recommended for their treatment. Additionally, laboratory data reveals that around 4% of global cases of MDR-TB can be traced back to Pakistan (Torumkuney et al., 2022).

Apart from bacterial infections, recent reports suggest that Pakistan is facing a growing issue of resistance to antimalarial drugs, with particular concern surrounding ACTs, which are the primary means of treating malaria. According to reports, there is a growing trend of resistance to antiviral medications employed in the treatment of hepatitis C, which is prevalent in the nation. The issue of AMR in Pakistan demands a synchronized approach involving healthcare professionals, policymakers, and the general populace. Enhancing monitoring, advocating for sensible usage of antibiotics, carrying out efficient infection management protocols, and allocating resources and efforts to healthcare infrastructure are essential measures that could be employed to manage AMR within the nation. Furthermore, raising consciousness and educating both healthcare professionals and the general public can prove to be crucial in managing the transmission of AMR. In Asia, AMR poses a major risk to public health due to the prevalent resistance observed among various bacterial species and contagious ailments. Efforts to tackle the issue of AMR in the region can only be successful through a united global approach. This includes enhancing surveillance, minimizing unwarranted use of antibiotics, producing novel antimicrobial treatments, and raising awareness and educating healthcare professionals and the general public. To enhance the prevention and management of infectious illnesses as well as AMR, investing in healthcare infrastructure and resources within the region is crucial (Roberts et al., 2022).

### The global predicament presented by the rise of antimicrobial resistance and the existence of AMR strains in Australia

Australia shows lower incidences of AMR in comparison to other global regions. However, there are apprehensions regarding the increasing prevalence of resistance in bacterial pathogens, namely MRSA and *Enterobacteriaceae*. Australia faces a major challenge regarding public health due to the emergence of AMR in multiple bacterial species, as evidenced by the significant rates of resistance. The issue of AMR in Australia is worsened by various factors including the unsuitable utilization of antibiotics, inadequate infection control measures, and traveling across borders. According to reports, there are significant levels of antibiotic resistance in various types of bacteria found in Australia, such as *Escherichia coli*, *Klebsiella pneumoniae*, and *Staphylococcus aureus*. Specifically, there is a growing concern for public health in the country due to the emergence of MRSA, which has been reported to have high levels of resistance in numerous hospitals and healthcare facilities (Wozniak et al., 2022). In Australia, there is a noteworthy concern regarding MDR-TB due to the high frequency of resistance observed toward both primary and secondary antibiotics employed for the management of this respiratory disease. According to reports, about 1.8% of recently detected TB cases in the nation exhibit resistance toward one of the primary antibiotics, while Australia contributes to approximately 7% of global cases of MDR-TB. Besides bacterial infections, it has been observed that antimalarial drugs like ACTs are also becoming less effective in Australia, where they are the primary medication to treat malaria. According to recent reports, there has been a surge in the prevalence of drug-resistant strains of hepatitis C, a disease that is widely spread across the region. To tackle the issue of AMR in Australia, a collective endeavor is needed involving healthcare practitioners, policymakers, and the general populace. To control AMR in the country, crucial measures that can be implemented include enhancing surveillance, advocating for rational utilization of antibiotics, enforcing effective infection control practices, and allocating resources toward healthcare infrastructure. Moreover, raising awareness and imparting knowledge to both healthcare professionals and the general public could be a crucial factor in managing the transmission of AMR (Cooper and Okello, 2021).

### The global repercussions stemming from antimicrobial resistance and the prevalence of antimicrobial-resistant species in Antarctica

Due to the low level of human presence in Antarctica, there is little data regarding the prevalence of AMR in the region. Nonetheless, research has revealed the existence of AMR genes in bacterial communities situated in Antarctica, underscoring the likelihood of resistance proliferation in this anomalous landscape. A research project gathered bacterial specimens from soil, water, and excrement samples taken from penguins and seabirds situated in the Antarctic Peninsula vicinity. A variety of antibiotics, such as tetracycline, ampicillin, and streptomycin, were used to determine the resistance levels of the isolates. The research revealed that every single isolate demonstrated resistance to at least one antibiotic, with tetracycline resistance prevailing extensively (Hwengwere et al., 2022). A different research unveiled the discovery of bacteria that are resistant to multiple drugs in a lake located in Antarctica. Various antibiotics, including ones such as ciprofloxacin, tetracycline, and amoxicillin, were discovered to have no effect on the bacteria due to their resistance. While the investigations imply the possible presence of AMR in Antarctica, it should be emphasized that the number of individuals residing in this area is limited, thereby reducing the likelihood of the expansion of drug-resistant microorganisms. Nonetheless, the occurrence of antimicrobial resistance in this distinct setting emphasizes the universal magnitude of this issue and the demand for ongoing supervision and observation of antimicrobial resistance on a global scale. Although there is insufficient data on the extent of antimicrobial resistance in Antarctica, recent research has revealed the possibility of the existence of resistant bacteria in this area. More comprehensive investigation and observation are required to gain a thorough comprehension of the scope and consequences of AMR in this distinct setting (Depta and Niedźwiedzka-Rystwej, 2023).

### Tactics for mitigating antimicrobial resistance (AMR) in nations with limited economic resources

It can be difficult to prevent the emergence of antimicrobial resistance (AMR) in low-income nations where factors like insufficient access to clean water, unsatisfactory sanitation and hygiene practices, insufficient regulatory oversight, and limited healthcare resources exist. Nevertheless, various preventive methods are available for low-income nations to tackle AMR. Enhance the prevention and management of infections through practicing fundamental hygiene measures such as thoroughly washing hands, sanitizing surfaces, and appropriately disposing of waste. Equipping medical personnel with PPE while offering training can play a significant role in curtailing the transmission of diseases. Encourage prudent utilization of antibiotics by means of instructing healthcare professionals and society on the proper application of antibiotics, while also discouraging the excessive and unwarranted consumption of these medications. Smartly increasing the accessibility of vaccines can effectively decrease the demand for antibiotics by preventing infections from occurring in the first place (Khan et al., 2020).

One of the key areas to focus on is the reinforcement of healthcare systems. This can be achieved by enhancing the capacity of laboratories, bettering surveillance systems to detect and report infections, and making sure that essential medicines are readily accessible. Create and execute plans for the responsible use of antibiotics known as antimicrobial stewardship programs. Such programs can effectively promote the appropriate and limited use of antibiotics. Their assistance can also diminish the emergence of antibiotic resistance through the endorsement of restricted-range antibiotics while restricting the usage of wide-ranged antibiotics (Khan et al., 2020). It is crucial to enhance the knowledge and understanding of the general public regarding the significance of preventing the spread of AMR and their contribution in reducing its propagation. It is necessary to promote the advancement of research and development in order to ensure that low-income countries have the opportunity to obtain novel antibiotics and other types of antimicrobial treatments. To guarantee these nations’ access to efficient remedies, governments, and international associations can endorse the research and development initiatives. Introducing these preventive measures can aid in mitigating the dissemination of AMR and safeguard the potency of present antibiotics. Achieving these objectives will necessitate a long-term commitment from governments, healthcare providers, and the general population (Sulis et al., 2022).

### Cutting-edge vaccination strategies devised to combat the challenge posed by antimicrobial resistance

The rise of AMR has resulted in an increased demand for novel and advanced vaccine technologies in order to tackle the proliferation of resistant bacteria. Several potential vaccine technologies are currently under investigation, showing promising results. Conjugate vaccines enhance immune responses by utilizing bacterial polysaccharides that are linked to carrier proteins. These vaccines have effectively thwarted infections caused by *Streptococcus pneumoniae* and *Haemophilus influenzae*, two key drivers of antimicrobial resistance (Rosini et al., 2020). Conjugate vaccines show great potential in the fight against AMR. Bacterial pathogens are targeted with antibodies generated by boosting immune responses through the use of carrier proteins and bacterial polysaccharides. The effectiveness of conjugate vaccines in the prevention of infections caused by two major AMR-triggering agents, *Streptococcus pneumoniae* and *Haemophilus influenzae*, has been proven. Conjugate vaccines offer a significant benefit as they can precisely aim at the particular strains of bacteria responsible for causing infections, rather than indiscriminately targeting the entire bacterial species. The reason for this is that the vaccine’s polysaccharide element is tailored to the bacterial strain, while its carrier protein element boosts the immune system’s reaction (Zhu et al., 2021). Conjugate vaccinations have exhibited remarkable efficacy in averting *pneumococcal* malady, instigated by *Streptococcus pneumoniae*. Pneumococcal illness is a significant reason for illness and death globally as it can result in severe conditions such as pneumonia, sepsis, and meningitis. The efficacy of conjugate vaccines in decreasing the frequency of pneumococcal disease, particularly in children, has been demonstrated to be tremendously high. Despite the potential benefits of conjugate vaccines in combating AMR, there are certain hindrances that need to be addressed. A major obstacle is the constant requirement for vaccine updates in order to keep pace with the changing bacterial strains. Another obstacle is the high expenses associated with creating and manufacturing these vaccines, making them impractical to pursue in certain environments. Although there are difficulties, conjugate vaccines still hold great potential to address antimicrobial resistance. Ongoing investigation and improvement in this field will be pivotal in combating AMR and averting bacterial infections (Rosini et al., 2020).

Vaccines in the form of virus-like particles (VLPs) imitate the structure of viruses without carrying any genetic material and are created through self-assembly. The visualization of antigens from bacteria on VLPs has the ability to trigger a robust immune response. VLPs have demonstrated efficacy in building immunity against the human papillomavirus (HPV) and hold potential for vaccine development against other pathogens that lead to AMR. VLP vaccines employ non-infectious particles resembling viruses to prompt the immune system to combat certain pathogens. Vaccines based on VLP have demonstrated potential in fighting AMR by directing their focus on bacterial pathogens. The viral protein coat of VLP vaccines is modified to showcase bacterial antigens, thereby prompting a defensive reaction from the host’s immune system. VLPs are deemed safer and more stable than conventional vaccines due to their non-infectious nature and lack of genetic material. Preclinical studies have revealed promising outcomes for VLP vaccines in addressing AMR. Vaccines for *Salmonella*, *Escherichia coli*, and *Clostridium difficile*, which are significant contributors to AMR, have been produced using VLP technology. In various animal models, it has been demonstrated that these vaccines are capable of producing an immune response and safeguarding against bacterial contamination. VLP vaccines have an edge in that they can be generated through recombinant DNA technology, enabling the speedy and flexible manufacture of vaccines. In the fight against AMR, investing in the creation of new vaccines can be a costly and lengthy process. Therefore, it is crucial to prioritize this effort. Nonetheless, there are still certain obstacles linked with the creation and utilization of VLP vaccines combating AMR. A major obstacle involves determining suitable bacterial markers to incorporate into the vaccination. Another hurdle to overcome is the possibility of the bacterial pathogen developing mechanisms to avoid the immune response, which can hamper the vaccine’s efficiency. In general, vaccines utilizing VLP present a hopeful solution for addressing the issue of AMR. It is crucial to carry on with further research and development in this field to effectively combat AMR and avert the spread of bacterial infections (Tagliabue and Rappuoli, 2018).

The method of DNA vaccines involves the use of plasmids that contain the genetic information for antigens, ultimately triggering an immune response. These vaccines possess stability and simplicity in production, making them a desirable choice for vaccine creation. Animal research has indicated that DNA vaccines are efficacious in combating bacterial illnesses including *Salmonella* and *Escherichia coli*. DNA immunizations possess promising capabilities of addressing AMR by directing their focus toward bacterial pathogens. In the DNA vaccines aiming to combat AMR, the plasmid employed includes genes that encode the desired bacterial antigen(s). Once the plasmid is introduced into the host’s system, the cells incorporate it and generate the antigen, leading to the activation of the immune system in response to the bacterial pathogen (Micoli et al., 2021). DNA vaccines are regarded as a safe and durable option that can be manufactured at a comparatively rapid pace and low cost. Many DNA vaccines aimed at combating AMR have undergone development and testing on animal models. An instance of a DNA vaccine that was effective against deadly MRSA infection in mice has been demonstrated. In animal models, encouraging outcomes have been demonstrated by the experimentation of DNA vaccines targeted toward battling *Streptococcus pneumoniae* and *Pseudomonas aeruginosa*. DNA vaccines have the benefit of being conveniently adaptable for the purpose of targeting various bacterial antigens, which facilitates the creation of vaccines for a broad spectrum of bacterial pathogens. Furthermore, the possibility of DNA vaccines offering extended safeguards against bacterial contagion is present since the recipient generates the antigen consistently. Despite the potential benefits of DNA vaccines in addressing AMR, there are still obstacles that need to be overcome. An issue that arises is the chance of a weakened immune system in certain people, leading to reduced efficacy of the vaccine. A further complication lies in effectively administering the vaccine, as it is crucial for the plasmid to target the correct cells to demonstrate its efficacy. In general, the use of DNA vaccines shows great potential in the fight against antimicrobial resistance (AMR). The ongoing investigation and improvement in this field will play a vital role in combating AMR and averting bacterial infections (Tagliabue and Rappuoli, 2018).

Live attenuated vaccines employ weakened versions of pathogens to trigger an immune response without inducing illness. The vaccines have demonstrated efficacy in averting infections triggered by measles, mumps, rubella, and varicella. The development of live attenuated vaccines for bacterial diseases is still in its infancy. Live attenuated vaccines utilize a debilitated or changed form of the pathogen to stimulate an immune reaction without inducing illness. Live attenuated vaccines have been studied as a potential solution for addressing bacterial pathogens that have become resistant to various antibiotics in the context of AMR. A live attenuated vaccine that targets AMR is exemplified by the *Salmonella* vaccine that has been attenuated (Seo and Seong, 2022). This vaccine employs a strain of *Salmonella* that has undergone genetic modification to lower its severity, and it has been weakened to ensure safety. Animal models have demonstrated that the vaccine effectively prevents *Salmonella* infection when given orally. One illustration is a vaccine containing weakened *A. baumannii* bacteria, which is intended to decrease the severity of infections caused by the multi-resistant bacterial pathogen. A modified strain of *A. baumannii* is incorporated into the vaccine. The virulence of *baumannii* has been reduced through attenuation. The vaccine demonstrated efficacy in safeguarding against *A. baumannii* in studies involving animals. Live weakened vaccines can offer extensive defense against various strains of a disease-causing microorganism. Despite the benefits of utilizing live attenuated vaccines against AMR, there are certain obstacles involved. A potential obstacle lies in the possibility of the vaccine strain reverting back to its dangerous form and resulting in illness. Moreover, live attenuated vaccines might not be appropriate for administering to people with compromised immune systems since the vaccine variation has the potential to provoke sickness in such individuals. In general, utilizing live attenuated vaccines appears to be a promising approach in the fight against antimicrobial resistance. The ongoing progress and innovation in this field will play a crucial role in combating AMR and fending off bacterial infections (Micoli et al., 2021; Seo and Seong, 2022).

Adjuvanted subunit vaccines employ certain substances that are incorporated into the vaccine in order to improve the immune system’s response. Adjuvanted subunit vaccines employ tiny fragments of the disease-causing agent in order to prompt an immune reaction. These vaccines have shown efficacy in preventing infections caused by human papillomavirus and hepatitis B virus and can be effective in creating vaccines against other pathogens that lead to antimicrobial resistance. Adjuvanted subunit vaccines belong to the category of vaccines that employ specialized segments of a pathogen, namely proteins or polysaccharides, in order to stimulate an immune reaction. The utilization of adjuvanted subunit vaccines has been investigated as a possible approach to address bacterial pathogens that have acquired resistance to various antibiotics, in the realm of AMR. A case in point of a subunit vaccine strengthened with an adjuvant to combat AMR is the 4CMenB injection, designed to counteract the *Neisseria meningitidis* serogroup B, which is a type of bacterial infection causing meningitis and sepsis. The immunization is made up of a combination of four parts which comprises three genetically engineered proteins and a single capsular polysaccharide. Additionally, this vaccine has been enhanced with the inclusion of aluminum hydroxide (Buchy et al., 2020). This refers to strains of meningitis-causing bacteria of the B serogroup that exhibit resistance to various antibiotics. A further instance pertains to a vaccination aimed at combatting *Staphylococcus aureus*, a bacterium that commonly exhibits antibiotic resistance. The vaccine employs a blend of polysaccharide and protein antigens, and it is fortified with alum and monophosphoryl lipid A. In studies conducted on non-human subjects, the vaccine demonstrated the ability to safeguard against *Staphylococcus aureus*. Adjuvanted subunit vaccines offer the benefit of being customizable to selectively target particular parts of a pathogen. This can enhance their effectiveness and minimize the occurrence of adverse reactions. Moreover, enhanced subunit vaccines supplemented with adjuvants are perceived as secure and can be given to people who have impaired immune systems. On the other hand, the utilization of adjuvanted subunit vaccines against AMR comes with some difficulties. A difficulty lies in the fact that certain vaccines may not offer a comprehensive defense against various strains of an infectious agent since they focus on particular elements. Moreover, the use of adjuvanted subunit vaccines may necessitate a series of vaccinations to attain adequate immunity, which can elevate the expense and intricacy of vaccination initiatives. In general, using adjuvanted subunit vaccines appears to be a hopeful approach to addressing antimicrobial resistance. Sustained exploration and innovation in this field will play a crucial role in combatting AMR and thwarting bacterial infections (Marianne et al., 2022).

### Sophisticated approaches addressing AMR by harnessing the power of RNA interference through small interfering RNA and shRNA

SiRNA and shRNA are RNA molecules capable of selectively targeting and inhibiting the expression of particular genes. SiRNA and shRNA technologies have been investigated as a viable approach to combat AMR by diminishing the expression of antibiotic resistance-related genes in bacterial pathogens. An instance of employing siRNA and shRNA methods in tackling AMR involves focusing on genes responsible for creating beta-lactamases enzymes that can disintegrate beta-lactam antibiotics like penicillin and cephalosporins. By suppressing the activity of these genes, siRNA and shRNA can enhance the potency of beta-lactam antibiotics against antibiotic-resistant bacterial strains (Ranasinghe et al., 2022).

An additional instance pertains to the selective targeting of genes responsible for the efflux pumps found in bacterial pathogens. These pumps expel antibiotics, leading to diminished antibiotic efficacy. The vulnerability of bacterial pathogens to antibiotics can be increased through the reduction of gene expression by means of siRNA and shRNA. The ability to target individual genes, offered by siRNA and shRNA technologies, is beneficial in a way that it reduces the probability of off-target effects and enhances the treatment’s effectiveness (Alshaer et al., 2021). Besides, siRNA and shRNA can be specifically formulated to target several genes simultaneously, thus enhancing their efficacy in combatting resistant bacterial strains. Nevertheless, there exist certain obstacles that are related to utilizing siRNA and shRNA methodologies to combat AMR. A difficulty lies in transporting these molecules to the site of the infection since they undergo quick degradation by nucleases present in the bloodstream. Moreover, siRNA as well as shRNA have the potential to exhibit off-target outcomes, which can cause inadvertent outcomes including gene suppression that are critical for the regular functioning of cells (Mahmoodi Chalbatani et al., 2019).

### The role of nanomedicine in the battle against antimicrobial resistance

The application of nanoparticles and other nanoscale materials for the detection and cure of illnesses is known as nanomedicine. Nanomedicine has been investigated as a promising approach to combat bacterial infections that have become resistant to conventional antibiotics in the context of AMR. One strategy involves utilizing nanoparticles as a vehicle for dispensing antibiotics directly to the precise site of infection. Enclosing antibiotics in nanoparticles has the potential to enhance their stability and extend their duration of action, thereby enhancing their capacity to combat antibiotic-resistant bacterial strains. Moreover, it is conceivable to customize nanoparticles to aim for particular types of bacteria or cellular pathways, thereby enhancing their precision and lessening the possibility of unintended effects. A different way to combat bacterial growth or rupture of bacterial membranes involves the utilization of nanomaterials. Silver nanoparticles have demonstrated the ability to counteract bacterial activity across multiple bacterial strains, suggesting their promising efficacy as a viable alternative to conventional antibiotics (Eleraky et al., 2020). Additional forms of nanoparticles, such as liposomes and dendrimers, have been investigated for their ability to combat bacteria. Apart from its possible therapeutic uses, nanomedicine has the potential to contribute to the advancement of novel diagnostic equipment for AMR (Seil and Webster, 2012). One potential application of nanoparticles is their utilization in identifying particular bacterial species or genes that contribute to antibiotic resistance in clinical samples. This could aid in making informed decisions about treatment and lessen the likelihood of antibiotic resistance developing. Despite the potential benefits, utilizing nanomedicine to combat AMR also poses certain obstacles that must be considered. An obstacle that exists regarding nanoparticles is their likelihood to gather within the surroundings which may cause non-target bacteria to develop resistance. Furthermore, a complete understanding of the safety implications of nanomedicine over time is still lacking, necessitating further study to evaluate any possible hazards related to their application (Coates et al., 2020).

### Harnessing clinical artificial intelligence as a potent tool in the fight against antimicrobial resistance

The use of AI could have a major impact in combating the issue of antimicrobial resistance (AMR). Using data analysis of past cases, AI has the ability to forecast the probability of antimicrobial resistance. By creating models that take into account different elements such as the characteristics of the bacteria, patient information, and patterns of antibiotic use, it becomes possible to anticipate and identify which antibiotics are the most probable to work effectively (Lau et al., 2021). The expedited designing of fresh antibiotics may be aided by the application of AI technology. By utilizing massive collections of chemical substances and their characteristics, machine learning algorithms can detect likely antibiotic prospects. One outstanding application of AI is in the designing and enrichment of antibiotic molecules, leading to decreased expenses and time required for conventional drug discovery procedures. AI technology can assist clinicians in real-time decision-making to optimize the deployment of antibiotics and improve their usage. With the help of AI algorithms, patient information can be examined to suggest the most suitable antibiotics and recommended amounts based on their medical condition and resistance levels. AI has potential applications in monitoring and supervising AMR through tracking and surveillance techniques. Sophisticated machine learning algorithms have the capacity to examine extensive collections of bacterial genomes and detect novel patterns of resistance, thereby providing valuable insights for addressing public health interventions. The use of artificial intelligence allows for tailored treatment of bacterial infections by taking into account the patient’s unique traits including their genetics, immune system, and microbiome, alongside the particular bacterial strain and its resistance patterns. This approach is known as precision medicine (Ali et al., 2023).

Artificial intelligence technology is used in clinical settings to combat antibiotic-resistant and multi-drug-resistant strains of microorganisms. Advanced technology in the form of clinical AI can make a crucial contribution to combatting the issue of AMR and countering the growth of MDRs. AI technology has the potential to aid in the detection of bacteria that are difficult to treat, anticipate patterns of antimicrobial resistance, and propose suitable treatment choices. Machine learning algorithms are an instance of AI technology that enables the interpretation of substantial quantities of patient data, thereby recognizing patterns and trends regarding AMR. This data can provide valuable insights to healthcare professionals in selecting the best possible treatment alternatives for their patients. A different illustration pertains to the creation of diagnostic tools that utilize AI to detect the existence of AMR and MDR species with speed and precision. Using these tools can effectively shorten the duration required for infection diagnosis, thereby facilitating prompt initiation of suitable treatment, resulting in an enhanced patient outcome (Lv et al., 2021).

Artificial intelligence has the potential to aid in the creation of novel antimicrobial treatments by estimating the efficacy of potential chemical compounds and revealing fresh objectives for medication innovation. Furthermore, artificial intelligence-based monitoring systems have the ability to oversee the diffusion of antibiotic-resistant and multidrug-resistant organisms and can swiftly detect potential risks as they arise. One can utilize this data to educate the public and shape government actions in order to curb the emergence and transmission of AMR. It’s crucial to acknowledge that incorporating AI into clinical environments demands meticulous examination of ethical and legal ramifications, alongside apprehensions regarding privacy of data. It is crucial to guarantee the ethical and transparent deployment of AI technology, together with safeguarding patient data (Lv et al., 2021; Ali et al., 2023).

### Cutting-edge CRISPR-based antimicrobials, bacteriophage therapies, and immunotherapies to confront antimicrobial resistance on the horizon

The issue of antimicrobial resistance poses a rising danger to worldwide health, and it is evident that innovative methods are necessary to tackle this issue. There are innovative methods for addressing AMR that may be implemented in the future. CRISPR-mediated antimicrobials employ gene editing tools that target and specifically eliminate particular types of bacteria. Scientists are investigating CRISPR-based antimicrobials as a possible substitute for antibiotics (Bikard and Barrangou, 2017). Bacteriophages, which are viruses that have the ability to infect and cause the death of bacteria, produce enzymes known as phage-derived enzymes. A group of scientists are exploring the possibility of using endolysins and depolymerases, which are enzymes derived from phages, as a substitute for antibiotics. The use of nanoparticle-based treatments involves engineering nanoparticles that have the ability to selectively eliminate bacteria. Scientists are investigating the possibility of substituting antibiotics with nanoparticles, specifically silver nanoparticles (Aslam et al., 2020). Scientists are exploring methods to manipulate the microbiome as a means of averting and curing bacterial infections. One possible approach is to introduce bacteria that can outcompete harmful ones, or modify the microbiome’s environment to reduce its suitability for these pathogens. Researchers are investigating the viability of immunotherapies, including monoclonal antibodies, as a possible substitute for antibiotics. These treatments may enhance the immune system’s capacity to combat bacterial infections. By utilizing artificial intelligence, the process of drug discovery can be expedited through the analysis of immense amounts of chemical compounds data and making predictions on the likelihood of their effectiveness. Scientists are investigating the potential of using artificial intelligence to discover novel antibiotics and other antimicrobial treatments (Mayorga-Ramos et al., 2023).

## Conclusion

**Figure 1:**
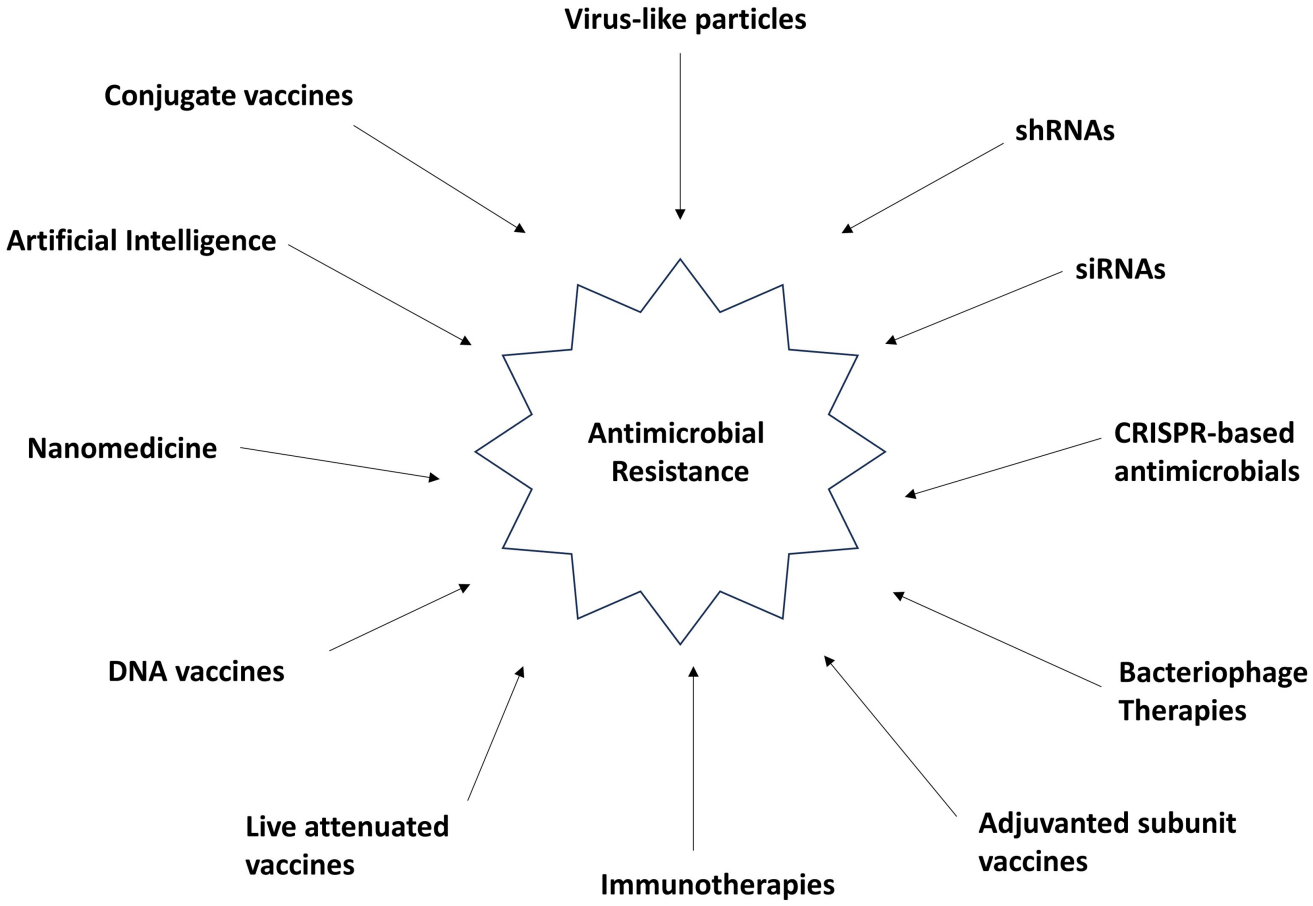
Strategies against multiple drug-resistant superbugs.

The formidable challenge of antimicrobial resistance (AMR) stands as a global imperative that transcends borders, impacting every corner of our interconnected world. To confront this looming menace, a united, worldwide collaboration is not just essential but an ethical obligation. Our response to AMR necessitates a comprehensive, multifaceted approach, including heightened surveillance, the development of innovative antibiotics and alternative therapies, and widespread education and awareness efforts among healthcare professionals and the public alike (Ibrahim et al., 2021a,b, 2022; Ismail et al., 2023). The urgency of the situation is undeniable, demanding immediate and decisive action on a global scale. Through international cooperation, we can safeguard the continued efficacy of antibiotics and antimicrobial treatments, ensuring their ability to combat bacterial infections and protect human well-being for generations to come. Promising avenues, such as bacteriophage therapy, vaccines, siRNA and shRNA technologies, nanomedicine, and artificial intelligence, hold the potential to bolster our arsenal against AMR, as shown in Figure 1. The responsible application of these innovations and the ongoing pursuit of scientific advancements are paramount. Together, we can navigate the complex terrain of AMR, preserve the integrity of antimicrobial agents, and pave the way for a future where bacterial infections are curable and global health thrives.

## Author contributions

US: Conceptualization, Data curation, Formal analysis, Funding acquisition, Investigation, Methodology, Project administration, Resources, Software, Supervision, Validation, Visualization, Writing – original draft, Writing – review & editing, Principal Investigator of study. RI: Data curation, Investigation, Validation, Visualization, Writing – review & editing. ZP: Data curation, Formal analysis, Funding acquisition, Investigation, Methodology, Project administration, Resources, Validation, Visualization, Writing – original draft, Writing – review & editing, Co-Principal Investigator of the study. MT: Investigation, Methodology, Project administration, Resources, Writing – review & editing. AS: Investigation, Methodology, Resources, Validation, Writing – review & editing. UA: Investigation, Methodology, Resources, Validation, Writing – review & editing. MF: Investigation, Methodology, Validation, Visualization, Writing – review & editing. SG: Methodology, Software, Validation, Visualization, Writing – review & editing. SN: Data curation, Formal analysis, Funding acquisition, Investigation, Methodology, Resources, Writing – review & editing. EN: Data curation, Funding acquisition, Investigation, Project administration, Visualization, Writing – review & editing. YW: Investigation, Methodology, Validation, Visualization, Writing – review & editing. MW: Investigation, Methodology, Validation, Visualization, Writing – review & editing. MN: Investigation, Methodology, Validation, Visualization, Writing – review & editing. IF: Investigation, Methodology, Validation, Visualization, Writing – review & editing.
